# Comprehensive Gene expression meta-analysis and integrated bioinformatic approaches reveal shared signatures between thrombosis and myeloproliferative disorders

**DOI:** 10.1038/srep37099

**Published:** 2016-11-28

**Authors:** Prabhash Kumar Jha, Aatira Vijay, Anita Sahu, Mohammad Zahid Ashraf

**Affiliations:** 1 Defence Institute of Physiology and Allied Sciences, Delhi, INDIA

## Abstract

Thrombosis is a leading cause of morbidity and mortality in patients with myeloproliferative disorders (MPDs), particularly polycythemia vera (PV) and essential thrombocythemia (ET). Despite the attempts to establish a link between them, the shared biological mechanisms are yet to be characterized. An integrated gene expression meta-analysis of five independent publicly available microarray data of the three diseases was conducted to identify shared gene expression signatures and overlapping biological processes. Using INMEX bioinformatic tool, based on combined Effect Size (ES) approaches, we identified a total of 1,157 differentially expressed genes (DEGs) (697 overexpressed and 460 underexpressed genes) shared between the three diseases. EnrichR tool’s rich library was used for comprehensive functional enrichment and pathway analysis which revealed “mRNA Splicing” and “SUMO E3 ligases SUMOylate target proteins” among the most enriched terms. Network based meta-analysis identified MYC and FN1 to be the most highly ranked hub genes. Our results reveal that the alterations in biomarkers of the coagulation cascade like F2R, PROS1, SELPLG and ITGB2 were common between the three diseases. Interestingly, the study has generated a novel database of candidate genetic markers, pathways and transcription factors shared between thrombosis and MPDs, which might aid in the development of prognostic therapeutic biomarkers.

Myeloproliferative disorders (MPDs) is a collective term to describe Polycythemia vera (PV), Essential thrombocythemia (ET) and Primary myelofibrosis (PMF) of which the pathogenesis is interconnected^1^. The MPDs have been classified as increased erythrocytes counts in PV, high platelet counts in ET and bone marrow fibrosis in the third disease PMF. However, the prevalence of PV and ET in human population is higher and well-studied as compared to the third one^2^. Venous thromboembolism being a multifactorial disease is the third most fatal cardiovascular complication after myocardial infarction and stroke and comes as a secondary complication to MPDs^3^. Evidences suggest that there are around 1.5 to 2 cases of PV and ET develop in a population of 100,000 annually^4^,^5^. Approximately 20% to 50% cases of PV and ET are identified with thrombotic complications in their vascular system. Although the incidences are more prevalent in PV, while for ET increasing age possesses a major risk factor for the progression of thrombogenecity. Remarkably, the thrombotic risk is well ascertained in PV and ET indicates that the intrinsic factors responsible for increase in platelet number and blood viscosity further contributing to the thrombotic events^6^. Epidemiological studies have recommended the pervasiveness of MPDs with thrombotic events but the knowledge regarding the hierarchical relationship between the biological pathways and shared mechanisms, if any, still lacks. Although the MPDs and thrombosis have different characteristics and appearance but thrombotic disorders could either be the result or a parallel secondary complication of MPDs^7^.

Microarray is a quantitative technique which facilitates the analysis of not only gene expression but the discovery of new drug targets, novel gene functions and new diagnostic alternatives in a single experiment simultaneously^8^. Gene expression analysis is the integral part of molecular genetics and functional genomics leading to understand and analyze global genomic patterns of different diseases. Meta-analysis is a systematic approach to study and combine different publically available dataset repositories to perceive shared molecular mechanisms of diseases, their risk factors or the effect of their treatment^9^. Literature evidences are available to correlate the robustness of the gene expression signatures through-out the genome for diseases of different types and origin^10^.

Merging multiple microarray datasets depends on powerful *in-silico* tools to manage and successfully interpret the complex data acquired from the study. Because different microarray studies are associated to different population, study design and diseases, it is very difficult to predict the accuracy of the method chosen for the analysis^11^. Meta-analysis provide enhanced statistical power, thereby obtaining more robust and reliable gene signatures and using Integrative meta-analysis of expression data (INMEX), a web based tool, facilitates careful data preprocessing and annotation to ensure that the data format and class labels are consistent across datasets. To address the differences in study design and platform usage, heterogeneity existing among microarray datasets, we applied the Effect size (ES) combination with Random Effect Modeling (REM) which takes both the direction and magnitude of gene expression changes into consideration to generate more biologically consistent results^12^.

Furthermore, the power of microarray meta-analytic techniques has also been well exploited to unearth the shared biological signatures between the related diseases and pathophysiological conditions by integrating the publically available microarray datasets^13^,^14^,^15^. In this study we have selected five eligible microarray datasets (based on the inclusion criteria) from public repositories for three different but correlated blood disorders; venous thrombosis (VT), ET and PV. This is the first time to our knowledge that a form of thrombosis is associated with MPDs implicating the common transcriptional signatures in healthy individuals versus patients.

## Results

### Selection of eligible microarray datasets

**Figure 1:**
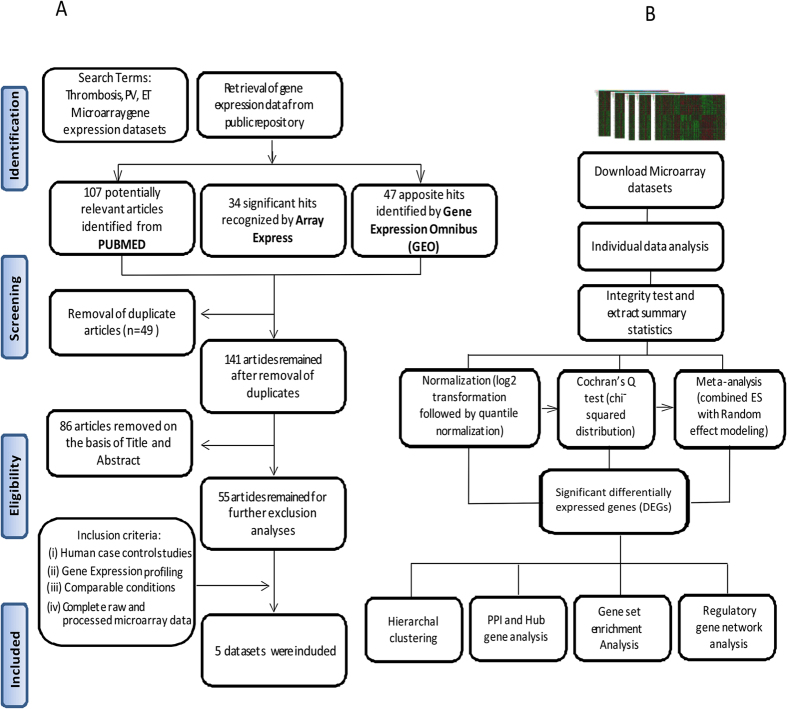
Workflow of microarray meta-analysis. (**A**) Selection process of eligible microarray datasets for meta-analysis of the shared signatures between thrombosis, essential thrombocythemia (ET) and polycythemia vera (PV), according to Prisma 2009 flow diagram. (**B**) Depiction of the flow chart of the process involved in integrated meta-analysis of the selected microarray datasets.

A total of 5 studies (accession number: GSE17078, GSE19151, GSE2006, GSE26049 and GSE47018) met the inclusion criteria (Fig. 1A) and were selected for meta-analysis, covering three types of coagulation related pathophysiology (2 datasets each for VT, ET and PV). Control/Patient datasets were considered with a collective number of 90/73, 29/25 and 27/60 controls/patients in VT, ET, and PV respectively. The datasets included in this meta-analysis were case/control studies where the controls were healthy individuals and are further defined similarly. Of note, all the datasets were generated using common microarray platform i.e. Affymetrix Human Genome U133A series. Only the studies included in which sample source were either whole blood or blood components, two studies GSE19151 and GSE26049 had whole blood, while blood outgrowth endothelial cells, platelets and peripheral blood CD34+ cells were the sample source of GSE17078, GSE2006 and GSE47018 respectively. Table 1 provides detailed information of each datasets and highlights the disease condition, sample type, references of the study and microarray platform used. It need to be mentioned that samples from GSE26049 were further separated into two subgroups with 19 and 41 patients (ET and PV respectively) and 21 common controls, these two subgroups were considered as individual datasets during meta-analysis using Integrative Meta-Analysis of Expression Data (INMEX), a web interface for integrative meta-analysis.

### Batch effect adjustment

The primary goal of the study was to identify the shared differentially expressed genes (DEGs) between thrombosis and MPDs using the selected datasets for meta-analysis, however, data integration is hindered by batch effects and efficient methods for batch effect removal are needed for integrative analysis. Before performing meta-analysis, INMEX takes care of reducing potential study-specific “batch effects” and thereby reducing confounding factors due to non biological variations. The pre-processed and normalized individual datasets were further subjected to the well-established ComBat procedures^16^. To compare the sample clustering patterns before and after applying the ComBat procedures, the results were visually examined using the principal component analysis (PCA). Multidimensional scaling of the datasets revealed that before application of the batch adjustment algorithm, each dataset clearly separated from all the others (“batch effect”), whereas after correction of batch effect, samples from all datasets were well intermixed (Figure S2).

### Identification of common Differentially Expressed Genes (DEGs) signatures among thrombosis and myeloproliferative disorders by meta-analysis

**Figure 2:**
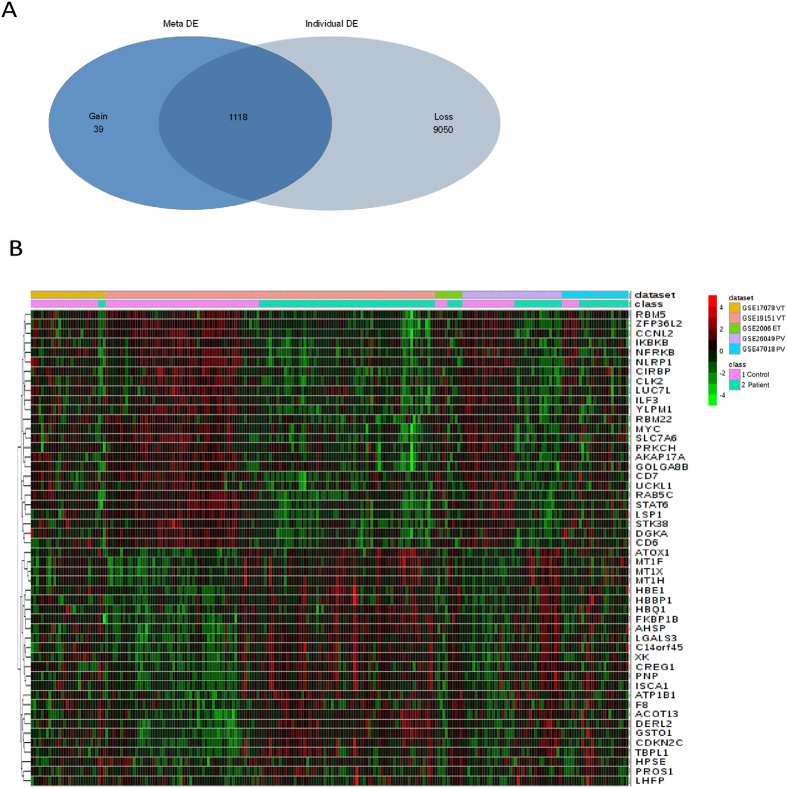
Gene expression pattern of the DEGs from meta-analysis. (**A**) Venn diagram of differentially expressed genes identified from the meta-analysis (Meta-DE) and those from each individual microarray analysis (Individual-DE). (**B**) Heat-map representation of expression profiles for the top 25 up- and 25 down-regulated DEGs obtained from meta-analysis. Clustering of selected genes on the heat-map was performed by hierarchical clustering algorithm using Euclidean distance measure. Class 1: Control; Class 2: Patient.

To identify a common transcriptional signature shared between thrombosis and MPDs (ET and PV), five microarray studies (Table 1) were analysed using INMEX. The overall meta-analysis workflow used in this study is shown in Fig. 1B. The processed data were loaded into INMEX webtool followed by the Cochran’s Q test Random Effect Modelling (REM) and ES (Effect Size) statistical analysis to find genes that were differentially expressed between patients and healthy controls across different studies. This statistical approach has the advantage of allowing the true effect size to vary from study to study by integrating unknown cross-study heterogeneities (i.e. due to non-biological heterogeneities). The implementation of this method is based on moderated effect size using the metaMA package^17^. From microarray meta-analysis we identified a total of 1,157 DEGs including 697 overexpressed and 460 underexpressed genes across the datasets under the significance threshold of adjusted *p-value* <0.05. As considered to be the advantage of meta-analysis 39 “gained” genes were additionally identified as DEGs in the meta-analysis with weak but consistent expression profiles across all five datasets, 9050 “loss” genes (Fig. 2A), (list shown in Supplementary sheet) were identified as DEGs in individual analysis but not in meta-analysis with inconsistent changes in expression profiles across different datasets, or large variations by different platforms or experimental errors. Figure 2B shows the heatmap of top 25 over and underexpressed genes across all datasets. The ZFP36 ring protein finger like 2 (ZFP36L2), LUC7 like (LUC7L) and NLR family, pyrin domain containing 1 (NLRP1) were among the most significantly overexpressed genes while, X-linked KX blood group (XK), Antioxidant 1 copper chaperon (ATOX1) and Protein S alpha (PROS1) were the most underexpressed genes across the five microarray datasets (Table 2). The complete list of DEGs is provided in supplementary sheet.

### Hub genes identification by network based meta-analysis

**Figure 3:**
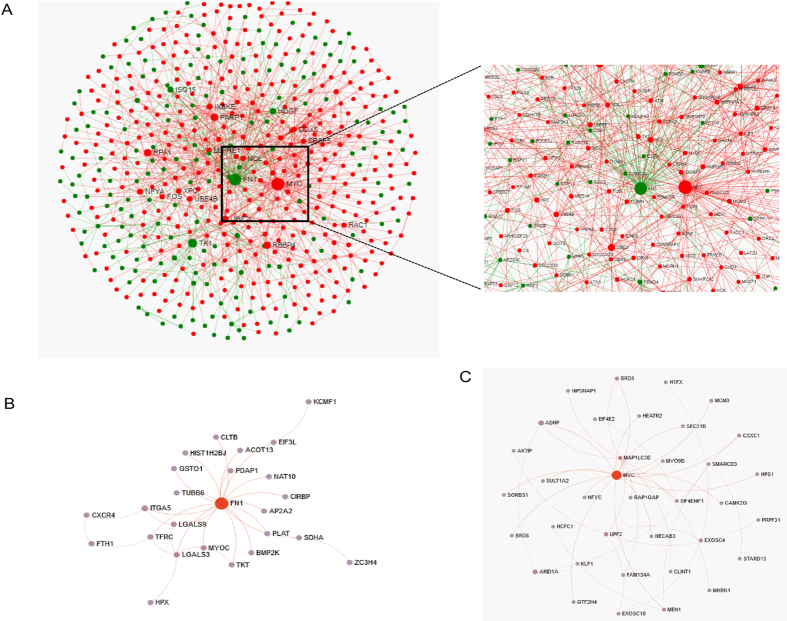
Network based meta-analysis of hub genes. (**A**) Zero-order interaction network of shared DEGs obtained from meta-analysis using force-directed algorithm with Fruchterman-Rengold layout; red nodes represents overexpressed and green nodes represents underexpressed DEGs. (**B**) PPI Subnetwork of most significant underexpressed DEG with its interacting partners. (**C**) PPI Subnetwork of most significant overerexpressed DEG with its interacting partners.

Network based meta-analysis was conducted to find out the key hub genes among the DEGs obtained from the meta-analysis of different datasets. NetworkAnalyst, a web based tool was implemented to generate a protein-protein interaction (PPI) network by integrating the InnateDB interactome with the original seed of 1,157 DEGs. An expanded PPI network was generated with 7,235 nodes representing the proteins and 24,928 edges representing the interaction between these proteins, however, for better visualization of the network “Zero order” interaction network was created with 555 nodes connected with 1,203 edges (Fig. 3A). MYC (myc protein) with the combined ES of 1.40 and adjusted *p-value* of 1.14E-06 and FN1 (Fibronectin 1) with the combined ES of −0.42 and adjusted *p-value* of 0.0097479 were found to be the most highly ranked hub genes among the overexpressed and underexpressed DEGs respectively. The most highly ranked nodes across the five datasets based on network topology measures were MYC (Betweenness centrality = 3371.02; Degree = 67) and FN1 (Betweenness centrality  = 3325.79; Degree = 67) followed by UBE2I (Betweenness centrality = 1215.46; Degree = 28) and IKBKE (Betweenness centrality = 688.90; Degree = 25), list of top ten hub genes based on network topology scores is shown in supplementary Table 1 (Table S1). Using the module explorer tool, most important modules were extracted as sub networks which included MYC (37 nodes and 48 edges) and FN1 (26 nodes and 34 edges). Both the modules are independent protein complexes with their interacting nodes likely to work collectively to perform biological functions (Fig. 3B and C).

### Gene set enrichment analysis for identification of overrepresented biological pathways and gene ontology terms

**Figure 4:**
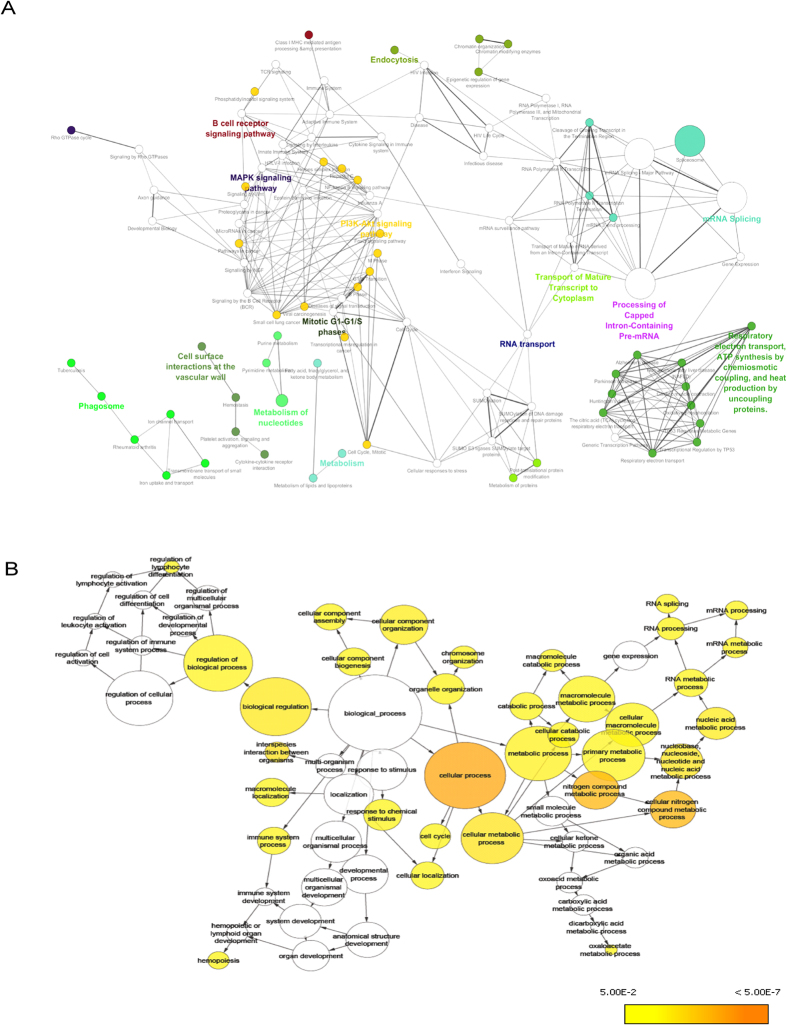
Overrepresentation of pathways and Gene Ontology categories in Biological Networks identified from meta-analysis. (**A**) Network representations of enriched pathway integrating KEGG and Reactome pathways on the DEGs gene list using ClueGO cytoscape plug-in. Hyper-geometric (right-handed) enrichment distribution tests, with a *p-value* significance level of ≤0.05), followed by the Bonferroni adjustment for the terms and leading term groups were selected based on the highest significance. The node size and deeper color indicates greater significance of the enrichment. The pathways having adjusted *p-value* <0.05 are shown in the network. (**B**) Enrichment network of shared DEGs based on biological processes. Significantly overrepresented biological processes based on GO terms were visualized in Cytoscape. The size of a node is proportional to the number of targets in the GO category. The color represents enrichment significance— the deeper the color on a color scale, the higher the enrichment significance. *p-values* were adjusted using a Benjamini and Hochberg False Discovery Rate (FDR) correction.

For the analysis of overrepresented biological pathways and gene ontology (GO) terms associated with the differentially expressed genes, we performed gene set enrichment analysis using EnrichR tool using the list of DEGs (including over- and underexpressed). GO terms and biological pathways were significantly overrepresented in the gene list if they showed an adjusted *p-value* <0.05. Results for enriched biological pathways and gene ontology are shown in Table 3. DEGs in meta-analysis results were associated with the enriched pathways with adjusted *p-value* <0.05, including “Processing of Capped Intron-Containing Pre-mRNA (R-HSA-72203)”, “mRNA Splicing (R-HSA-72172)” and “SUMO E3 ligases SUMOylate target proteins (R-HSA-3108232)”. The most important GO terms associated with overexpressed genes included “RNA splicing (GO:0008380)”, “mRNA processing (GO:0006397)”, “cytosol(GO:0005829)” and “nucleoplasm (GO:0005654)”. To gain further insights of shared GO terms and biological pathways associated with the meta-analysis DEGs, we used two separate bioinformatic softwares in the integrative environment of cytoscape V3.1. Functionally grouped network of enriched biological pathway categories associated to KEGG and reactome pathway databases were generated for the DEGs using ClueGO V2.1.7, which facilitates the visualization of pathway interaction in the form of network (Fig. 4A). Using BinGO enrichment clusters of biological processes (GO) associated with DEGs of meta-analysis was generated with following groups among the most enriched terms: “cellular process”, “mRNA processing”, “biological regulation”, “immune system process” and “regulation of lymphocyte differentiation” (Fig. 4B).

### Identification of the transcription factors and regulatory kinases network upstream to the shared Differentially Expressed Genes obtained from meta-analysis

Expression2Kinase (X2K) bioinformatic tool was used to perform regulatory gene network analysis to identify upstream regulators responsible for observed patterns in gene expression meta-analysis studies. Emphasis was made to infer the most important transcription factors and protein kinases associated with the complete set of DEGs and rank these regulatory gene candidates rendering their involvement in the formation of regulatory complexes. List of top 10 ranked transcription factors and protein kinases are shown in supplementary Table 2 (Table S2). The interaction network was constructed between transcription factors, kinases and their intermediate proteins involved in formation of regulatory complex. Fli-1 Proto-Oncogene, ETS Transcription Factor (FLI1) and Hepatocyte Nuclear Factor 4, Alpha (HNF4A) were among the top transcription factor while Mitogen-Activated Protein Kinase 1/3 (MAPK1/3) and Homeodomain Interacting Protein Kinase 2 (HIPK2) were among the top protein kinases associated with the DEGs from the meta-analysis across the five datasets (Supplementary Table S2).

### Shared coagulation related gene signatures

To identify the coagulation related gene signatures, we briefly conducted biological process (Gene Ontology) analysis on the complete set of DEGs (both over- and underexpressed) using EnrichR tool. In the analysis “blood coagulation (GO:0007596)” with overlap (45/472) was observed. Table 4 depicts the expression pattern of top ten coagulation genes; Selectin P Ligand (SELPG), Carboxypeptidase B2 (CPB2), integrin, beta 2 (ITGB2), Protein kinase C, eta (PRKCH), Ras-related C3 botulinum toxin substrate 1 (rho family, small GTP binding protein Rac1) (RAC1), 3-phosphoinositide dependent protein kinase-1 (PDPK1), Guanine nucleotide binding protein (G protein), alpha inhibiting activity polypeptide 2 (GNAI2) and Lysine (K)-specific demethylase 1A (KDM1A) were among the overexpressed while the expression of Coagulation factor II (thrombin) receptor (F2R) and protein S (alpha) (PROS1) were underexpressed. There was consistent expression of these genes across the five datasets as shown in supplementary sheet.

## Discussion

Thrombotic events are present in 20% to 50% of patients with PV and ET at the stage of diagnosis and involve complications in both major vessels and microcirculation. PV and ET are chronic myeloproliferative disorders, the benign clinical course of which can be complicated by thrombotic events. Thrombotic complications are characterized by microcirculatory disturbances and increased risk of arterial and venous thrombosis^18^,^19^,^20^,^21^,^22^. The mechanisms underlying the thrombotic events in these cases are still largely obscured and more importantly the number of large scale studies performed in this specific setting is very limited for identification of shared genetic markers responsible for the predisposition of the thrombosis in MPDs. Although a large quantity of data has been produced using microarray studies, the small sample size of these studies is a significant obstacle to the identification of common DE genes. A meta-analysis of multiple microarray datasets increases the sample size, making the identification of DE genes more reliable. However, previous microarray studies have typically focused on identifying factors specific to any of the three diseases and focused little on identifying genes and risk factors shared between thrombosis and MPDs^23^,^24^,^25^,^26^,^27^. Therefore, in this study, we attempted to identify common genes underlying thrombosis, PV and ET using gene expression meta-analysis of 5 publically available microarray data. To the best of our knowledge, this is the first such attempt in thrombosis research.

By jointly analyzing 5 published microarray gene expression datasets of thrombosis, PV and ET, we defined a common signature of a total of 1,157 DEGs including 697 overexpressed and 460 underexpressed genes across the datasets under the significance threshold of adjusted *p-value* <0.05 in all diseases compared to healthy controls. Interestingly, we identified 38 “gained” DEGs in this meta-analysis which were not discovered in the prior individual analyses (supplementary sheet). Among the top ten overexpressed DEGs, ZFP36L2 (ZFP36 ring finger protein-like 2) had the highest combined ES of 2.26; it is an RNA binding protein and functions as a molecular switch promoting early burst-forming unit-erythroid (BFU-E) self-renewal and a subsequent increase in the total numbers of colony-forming unit-erythroid (CFU-E) progenitors and erythroid cells that are generated^28^. Inflammation is one of the major contributor in the pathogenesis of thrombotic complications, evident from the overexpression of Nucleotide-binding, leucine-rich repeat, Pyrin domain containing 1 (NLRP1) which potentiates the peripheral immune response, caspase-1 activation involves the formation of a macromolecular complex termed as inflammasome^29^.

While Cyclin L2 (CCNL2) a regulatory protein involved in pre-mRNA splicing process has been shown to be upregulated in ET^30^ which is in agreement with the findings in present meta-analysis study. Among the underexpressed DEGs X-linked Kx blood group (XK) had the highest combined ES (−1.60); it controls the synthesis of the Kell blood group ‘precursor substance’ (Kx) and is involved in maintenance of hematopoietic systems, however, its direct role in thrombosis and MPDs is not known. Interestingly, downregulation of Protein S alpha (PROS1) an essential physiological anticoagulant has been established to be one of the important markers for thrombosis^31^,^32^ although no direct evidence of the role of PROS1 in relation to MPDs have been ascertained. Perturbation in the expression of heparanase (HPSE) an endo-beta-glucuronidase that is capable of cleaving heparan sulfate side chains of heparan sulfate proteoglycans on cell surfaces and extracellular matrices have been widely associated with thrombosis, PV and ET^33^,^34^. Therefore, our results are consistent with previously published data for each of the three disorders, but for the first time to our knowledge, we formally show their shared genetic signatures.

The emerging tools of network biology offer a platform to systematically investigate the molecular complexity of a particular disease, leading to the biomarker discovery and identification of drug targets for the improvement in disease management^35^. Network-based meta-analysis from the original list of DEGs was conducted for the prioritization of the most important hub genes based on network centrality scoring. V-Myc Avian Myelocytomatosis Viral Oncogene Homolog (MYC) and FN1 were the most important hub genes among over and under-expressed genes respectively across five microarray studies. Of note, FN1 was identified as the unique marker from meta-analysis as the part of “gain genes” which was not identified as DEGs in the individual microarray studies. MYC is a multifunctional, nuclear phosphoprotein acts as a transcription factor and plays a role in cell cycle progression, apoptosis and cellular transformation. Mutation and overexpression of MYC gene have been reported as a risk factor for blast transformation and fibrotic progression in PV and ET^36^. Platelets play a key role in maintaining the fine balance between thrombosis and hemostasis, it has been established that pattern of c-MYC expression, is key to producing functional platelets from selected induced pluripotent stem cells (iPSC) clones^37^ pointing to the relation between thrombosis and ET, a chronic disorder related to the over production of thrombocytes. Fibronectin (FN1), a glycoprotein present in plasma, cell surface and in extracellular matrix is involved in cell adhesion and migration processes including embryogenesis, wound healing and blood coagulation via its interaction with various compounds: collagen, fibrin and actin. Significant reduction in the plasma levels of fibronectin has been reported due to expanded mononuclear phagocyte system present in the liver and spleen, reduced hepatic synthesis and the clearance of circulating immune complexes^38^. The balance between hemostasis and thrombosis relies on a well maintained adhesive response of blood platelets with coagulation factors and adhesion molecules. FN1 has been recently associated with platelet thrombus formation via its cell adhesion property^39^,^40^. Tissue plasminogen Activator (tPA) responsible for clot dissolution by mediating the activation of plasmin has a high affinity for FN1 forming the basis of “clot buster”^41^. Thus the downregulation of FN1 can be well linked to the fact that lack of clot dissolution potentiates the thrombotic pathophysiology.

In order to elucidate the role of DEGs obtained from the meta-analysis, we performed gene set enrichment analysis and pathway analysis using the comprehensive enrichment library of EnrichR platform on both the gene list of over and under expressed DEGs. Interestingly, the most enriched pathway and Gene Ontology (GO) term among the shared DEGs of meta-analysis were “Processing of Capped Intron-Containing Pre-mRNA (R-HSA-72203)”, “mRNA Splicing (R-HSA-72172)”, RNA splicing (GO:0008380)” and “mRNA processing (GO:0006397)”. Several studies in the recent past are in agreement with our findings as they have associated various Spliceosome complex genes including U2 Small Nuclear RNA Auxillary Factor 1 (U2AF2), pre-mRNA processing factor (PRPF), splicing factor 3 (SF3) subunits and Serine Arginine rich factors (SRSF) using whole exome/genome technologies in myelodysplastic syndromes and in other hematologic disorders^42^,^43^,^44^. Briefly to understand the association of the DEGs list to the most significant kinases and transcription factors, we conducted regulatory gene network analysis using X2K software. The mitogen-activated protein (MAP) kinases MAPK1, MAPK3, MAPK14 were amongst the most significant kinases associated with the DEGs. Besides other cells, MAP kinases are present in platelets also and are activated by various stimuli, such as thrombin, collagen, von Willebrand factor (VWF) and ADP. These molecules/stimuli have established role in thrombosis and MPDs,^46^. Among the most significant transcription factors associated with the DEGs were friend leukemia integration 1 (FLI1), Runt-related transcription factor 1 (RUNX1) and GATA-binding factor 1 (GATA1) which are established as hematopoietic transcription factors to be involved in hemostasis and MPDs especially platelet dysfunction^47^. FLI1 is a transcription factor, plays major role in megakaryopoiesis by influencing the expression of several genes and the expression of FLI1 was shown to be significantly high in MPDs^48^.

From the clinical standpoint, the tendency of perturbation in coagulation mechanism towards procoagulant state potentiates the risk of thrombosis which is one of the most exceptional characteristics of the myeloproliferative neoplasms, especially applying to PV and ET. Using differential gene expression meta-analysis, we found several coagulation related genes previously identified by alternative strategies, having a potential role in thrombosis and MPDs. Evidences suggest that the decrease in the level of natural anticoagulants including Protein S alpha (PROS1) and coagulation factor II (thrombin) receptors are associated with PV and ET patients with thrombosis and our data is in agreement with the study^49^. The overexpression of Selectin P Ligand (SELPLG) and Integrin beta 2 (ITGB2) are independently and significantly reported to be involved as the shared markers in the pathogenesis of PV and ET with thrombosis^50^,^51^. Although the role of other coagulation proteins including Protein kinase C, eta (PRKCH), Ras-related C3 botulinum toxin substrate 1 (rho family, small GTP binding protein Rac1) (RAC1), 3-phosphoinositide dependent protein kinase-1 (PDPK1), Guanine nucleotide binding protein (G protein), alpha inhibiting activity polypeptide 2 (GNAI2) and Lysine (K)-specific demethylase 1A (KDM1A) are reported to be involved in the pathophysiology of thrombosis, ET and PV individually, however, the exact mechanism of these genes sharing the role in all three diseases still requires in-depth research.

While the present study provides important insights into the shared genetic markers and pathways between thrombosis and MPDs at the molecular level, it would be sensible to highlight the strengths and limitations of our study. Firstly, heterogeneity and confounding factors may have distorted the analysis. Although we conducted individual normalization for different datasets, the heterogeneity of technical variations in individual studies cannot be removed completely. Second, there are differences in gene expression between tissues such as whole blood, platelets, CD34+ cells and blood outgrowth endothelial cells and confounding factor due to sample source variation remains the major limitation of our study as these effects are hard to assess due to the paucity of available datasets in human for the three diseases we included in this study. Although many sophisticated algorithms have been published in recent years, no single statistical method is optimal. However, batch effect adjustment, individual data preprocessing and normalization and the random effect model based on Cochrans’Q test was done to reduce the non-biological heterogeneities in the present study. Furthermore, the use of similar platform (Affymetrix) for generation of microarray data across all the five datasets adds to the strength of the study. Briefly, we used INMEX tool to perform meta-analysis, which uses one of the best statistical methods and has been used in several recent publications^52^,^53^,^54^.

In conclusion, this is the first report that provides biological insights on common gene expression signatures shared between thrombosis, PV and ET comprising many genes that have been previously related to one, two or each of the three diseases. Although there are previous individual gene expression microarray studies on each diseases and in combination, this study is the first one, to our knowledge, where data on these three specific disorders have been integrated, which allowed us to define common biological processes. In addition, our results strengthen the association between Thrombosis, PV and ET and provide insights into the molecular mechanisms underlying the modulation of coagulation cascade. Furthermore, this study emphasizes on the potential of network analysis as a powerful framework to gain insight into the most important hub genes underlying the shared pathophysiologies between the three diseases and to identify potential therapeutic targets and biomarkers of disease. Further, in-depth functional studies on these common genes may improve our understanding of the pathological processes of these diseases, which could have important implications for the prevention and treatment of thrombosis related complications in MPDs in general.

## Methods

### Identification and selection of eligible gene expression datasets for meta-analysis

We systematically mined PubMed database for microarray expression profiling. The following key words and their combinations were used: “Thrombosis, Polycythemia vera, Essential thrombocythemia, microarray, gene expression dataset”. In addition, publicly available microarray datasets by April 2016 were searched in two public repositories: NCBI Gene Expression Omnibus (GEO) (http://www.ncbi.nlm.nih.gov/geo/) and ArrayExpress database of the European Molecular Biology Laboratory–European Bioinformatics Institute (http://www.ebi.ac.uk/arrayexpress/) to ensure no relevant studies were missed. The following information was extracted from each identified study: GEO accession number, sample type, platform, number of cases and controls, references, and gene expression data (Table 1). Inclusion criteria were set and strictly followed for dataset selection: human case/control study, comparable conditions, untreated samples and availability of raw and processed data. Non-human studies, review articles and integrated analysis of expression profiles were excluded. We conducted this meta-analysis in accordance with the guidelines provided in the Preferred Reporting Items for Systematic Reviews and Meta-Analysis (PRISMA) guidelines published in 2009^55^. Figure 1A shows the complete workflow of eligible dataset selection.

### Batch effect adjustment and Individual data analysis

Batch effect correction option in INMEX tool was used to reduce potential study-specific batch effects, the pre-processed and normalized individual datasets were further subjected to the well-established ComBat procedures^16^. It uses Emperical Bayes methods designed to stabilize the expression ratios for genes with very high or very low ratios, stabilize gene variances by shrinking variances across all other genes, possibly protecting their inference from artifacts in the data. To compare the sample clustering patterns before and after applying the ComBat procedures, the results were visually examined using the principal component analysis (PCA) (supplementary Figure S2). Individual dataset preprocessing and normalization was done by log2 transformation and quantile normalization, each individual dataset was visualized in box plots to ensure identical distribution among the samples and identify potential outlier.

### Microarray meta-analysis

We conducted a microarray meta-analysis using Integrative Meta-Analysis of Expression Data (INMEX), a web interface for integrative meta-analysis^12^. All gene probes were converted to a common Entrez ID using the gene/probe conversion tool in INMEX. After matching all probes to a common Entrez ID, individual datasets were preprocessed using the log2 transformation and quantile normalization. Each individual dataset was visualized in box plots and Principal Component analysis (PCA) plots to ensure identical distribution among the samples. GSE26049 contains samples from ET and PV patients but common controls; therefore, these two subpopulations were treated as two different datasets during the analysis. Differential expression analysis was performed with INMEX for each dataset independently using adjusted *p-value* <0.05, based on the false discovery rate using the Benjamini–Hochberg procedure and moderated t-test based on the Limma algorithm^56^. For meta-analysis, data integrity was checked for all datasets, and the differential expression meta-analysis across diseases and healthy controls was carried out by Effect size (ES) combination which takes into consideration both the direction and magnitude of gene expression changes to generate more biologically consistent results. The random effect model^57^,^58^ was chosen over Fixed Effect Model (FEM) because there were significant cross-study heterogeneities based on the Cochrans’ Q^59^ test, Random effects model (REM) is based on combining the effect sizes (ESs) or changes of gene expression from different studies and obtaining an overall mean with a significance level of adjusted *p-value* <0.05. Heatmap visualization of a subset of 25 over and under-expressed genes from the meta-analysis was performed using the “Pattern extractor” tool from INMEX (Fig. 2B) the data for this heatmap normalized within each study before being pooled together. Figure 1B shows the overall steps in microarray meta-analysis.

### Network- based hub gene analysis

Network-based analysis was performed using NetworkAnalyst^60^ which is designed to support integrative analysis of gene expression data through statistical, visual and network-based analysis by taking the advantage of common functions for network topology and module analyses approaches. Briefly, the complete list of DEGs both over-expressed and under-expressed were uploaded into the web- based server of NetworkAnalyst and network construction was restricted to contain only the original seed proteins by selecting the zero order interactors to avoid “hairball effect” and to allow proper visualization of interaction network. To help identification of highly interconnected hub nodes, NetworkAnalyst provides two widely used network centrality topological measures-degree and betweenness centrality. The degree of a node is the number of connections it has with other nodes. The betweenness centrality measures number of shortest paths going through the node and nodes with the highest betweenness, control most of the information flowing in the network, representing the critical points of the network^61^. From the parent network the most important modules (sub networks) of over and under-expressed DEGs were extracted using the “module explorer” panel which is based on the well-established Walktrap algorithm based on random walks^62^.

### Functional gene set enrichment analysis of shared Differentially expressed genes (DEGs)

To discern the implication of shared DEGs on thrombosis and MPDs, we performed a functional analysis using the EnrichR platform^63^. This state-of-the-art web based software allows evaluation of annotations, significantly enriched in a gene list, with its extensive gene set libraries including Gene ontology^64^ and various pathway analysis libraries like Kyoto Encyclopedia of Genes and Genomes pathway (KEGG), Reactome pathway, Wikipathway, panther and biocarta Enriched pathway and gene ontology were selected with adjusted *p-value* <0.05. EnrichR platform used for gene set Enrichment Analysis in this study performs the *p-value* adjustment by Z-score permutation background correction on Fischer Exact Test *p-value* for large gene sets. It gives rank based ranking to the enriched pathways derived from running the Fischer Exact Test for many random gene sets in order to compute a mean rank and standard deviation from the expected rank for each term in the gene set library and finally calculating a Z-score to assess the deviation from the expected rank. For better visualization and interpretation of the biological significance of shared DEGs, we separately conducted the analysis using cytoscape v3.1^65^ plug-ins. Using ClueGO^66^ a user friendly Cytoscape plug-in to analyze interrelations of terms and functional groups in biological networks, we conducted pathway analysis by integrating KEGG and Reactome pathway on the DEGs gene list. We used enrichment (right-sided) hyper-geometric distribution tests, with a *p-value* significance level of ≤0.05, followed by the Bonferroni adjustment for the terms and the groups with Kappa-statistics score threshold set to 0.3, whileleading term groups were selected based on the highest significance (Fig. 4A). Using Biological Networks Gene Ontology tool (BiNGO)^67^, an open-source cytoscape plug-in, we verified which Gene Ontology (GO) biological process terms are significantly overrepresented in a set of DEGs by hyper-geometric test statistics, followed by Benjamini and Hochberg false discovery rate (FDR) correction (Fig. 4B).

### Expression2Kinases (X2K) analysis of regulatory gene networks

To gain further insights into the upstream regulation of gene expression of DEGs, we uploaded the complete list of shared DEGs formatted such that there is one Entrez Gene Symbol on each line with no dashes, spaces or special characters in Expression2Kinases (X2K) software^68^. Using the transcription factors and kinases module which make use of chip-X from ChEA^69^ database as background, we extracted ten most significant transcription factors and kinases based on Fischer Exact test *p-value* enrichment scoring. Regulatory network was created and visualized on cytoscape environment from the “graphml” file generated from the analysis (Supplementary Fig. S1). The network ensures that the protein network obtained during the network expansion must have properly connected nodes with edges; in case the enriched transcription factors and kinases are not connected, the path length is automatically increased so that there are more intermediate proteins to connect the transcription factors.

### Statistical analyses

The meta-analysis was performed using the web-based tool—INMEX. The effect size combination using the random effect model was used for meta-analysis. Effect size is a standardized difference defined as the difference between group means divided by its standard deviation (i.e. Z-score). An adjusted *p-value* of <0.05, based on the false discovery rate using the Benjamini–Hochberg procedure was used to select DE genes. For the functional enrichment analysis, significantly enriched GO terms in DEGs relative to the genomic background by GO function software packages were identified using Selected statistical test: Hypergeometric test (right-sided) and Selected correction: Bonferroni/Benjamini & Hochberg False Discovery Rate (FDR) correction. Significantly enriched pathways were identified using hypergeometric tests and an adjusted *p-value* ≤0.05 was applied as the cut-off value for statistical significance.

## Additional Information

**How to cite this article**: Jha, P. K. *et al.* Comprehensive Gene expression meta-analysis and integrated bioinformatic approaches reveal shared signatures between thrombosis and myeloproliferative disorders. *Sci. Rep.* **6**, 37099; doi: 10.1038/srep37099 (2016).

**Publisher's note:** Springer Nature remains neutral with regard to jurisdictional claims in published maps and institutional affiliations.

## Supplementary Material

Supplementary Information

Supplementary Sheet

## Figures and Tables

**Table 1:** Characteristics of individual studies included in the meta-analysis.

GEO accession no.	Disease	Samples (Ctl/Pt)	Sample source	Platform	Reference
[GSE17078]	Venous Thrombosis (VT)	(n = 30) 27/3	Blood Outgrowth Endothelial Cells	Affymetrix Human Genome U133A Array	70
[GSE19151]	Venous Thrombosis (VT)	(n = 133) 63/70	Whole Blood	Affymetrix Human Genome U133A 2.0 Array	71
[GSE2006]	Essential Thrombocythemia (ET)	(n = 14) 8/6	Platelets	Affymetrix Human Genome U133A Array	72
[GSE26049]	Polycythemia Vera (PV), Essential Thrombocythemia (ET)	(n = 81) 21/41 (PV)/19 (ET)	Whole Blood	Affymetrix Human Genome U133 Plus 2.0 Array	73
[GSE47018]	Polycythemia Vera (PV)	(n = 25) 6/19	Peripheral blood CD34+ cells	Affymetrix Human Genome U133A Array	74

GEO: Gene Expression Omnibus; GSE 26049 was further separated into two subgroups with 19 Essential Thrombocythemia and 41 Polycythemia Vera patients with 21 common controls, these two subgroups were considered as individual datasets during meta-analysis.

**Table 2:** Top 20 shared DEGs identified in the meta-analysis.

EntrezID	Gene symbol	Gene Name	Combined ES	Adjusted *p-value*
**Top 10 Over-expressed Genes**				
678	ZFP36L2	ZFP36 ring finger protein-like 2	2.2679	8.26E-06
55692	LUC7L	LUC7 like	1.7633	9.99E-05
22861	NLRP1	NLR family, pyrin domain containing 1	1.6398	2.28E-11
440270	GOLGA8B	Golgin A8 family member B	1.543	0.001225
5878	RAB5C	RAB5C, member RAS oncogene family	1.5252	0.010788
1606	DGKA	Diacylglycerol kinase alpha	1.4978	0.000458
9057	SLC7A6	Solute carrier family 7 member 6	1.4452	0.018486
81669	CCNL2	Cyclin L2	1.4413	0
55696	RBM22	RNA binding motif protein 22	1.4279	1.63E-05
923	CD6	CD6 molecule	1.4242	0.00429
**Top 10 Under-expressed Genes**				
7504	XK	X-linked Kx blood group	−1.6059	9.19E-08
475	ATOX1	Antioxidant 1 copper chaperone	−1.4773	1.99E-06
8804	CREG1	Cellular repressor of E1A stimulated genes 1	−1.4133	0.001229
5627	PROS1	Protein S (alpha)	−1.3574	0.000767
10855	HPSE	Heparanase	−1.336	0.022878
4501	MT1X	Metallothionein 1X	−1.3259	1.85E-06
9446	GSTO1	Glutathione S-transferase omega 1	−1.2905	0
4860	PNP	Purine nucleoside phosphorylase	−1.2799	0
2281	FKBP1B	FK506 binding protein 1B	−1.262	0.001107
51327	AHSP	Alpha hemoglobin stabilizing protein	−1.2479	1.30E-09

Genes were ranked based according to the Standardized difference, also known as effect size. The corresponding *p-value*s are adjusted, based on the false discovery rate using the Benjamini–Hochberg procedure used to select DE genes obtained in each meta-analysis. Combined ES: Combined Effect.

**Table 3:** Top enriched terms and biological pathways identified by functional analysis of the DEGs in the meta-analysis.

Enrichment Term	Pathway/Term ID	Overlap	GSEA library	Adjusted P-value
**Enriched Pathways**				
Processing of Capped Intron-Containing Pre-mRNA	R-HSA-72203	33/193	Reactome	0.00634
mRNA Splicing - Major Pathway	R-HSA-72163	25/134	Reactome	0.012207
mRNA Splicing	R-HSA-72172	25/144	Reactome	0.023195
SUMO E3 ligases SUMOylate target proteins	R-HSA-3108232	19/96	Reactome	0.029891
mRNA Processing	WP411	25/127	WikiPathways	0.015246
**Enriched Gene Ontology term**				
RNA splicing	(GO:0008380)	45/313	GO	0.011692
mRNA processing	(GO:0006397)	50/397	GO	0.018005
mRNA splicing, via spliceosome	(GO:0000398)	29/177	GO	0.018005
Cytosol	(GO:0005829)	221/2529	GO	0.003815
Nucleoplasm	(GO:0005654)	115/1051	GO	0.000197

Overlap: indicates the number of hits from the meta-analysis compared to each curated gene set library. Gene set functional analysis was performed using extended libraries of the EnrichR tool. Enriched terms and pathways were ranked based on the adjusted *p-value*. GO: gene ontology biological process; GSEA: Gene Set Enrichment Analysis.

**Table 4:** Top coagulation related genes across the different datasets of meta-analysis.

Gene	Gene name	Role	Fold change
SELPLG	Selectin P Ligand	Facilitates calcium-dependent interactions with E P and L-selectins, mediates rapid rolling of leukocytes over vascular surfaces during the initial steps in inflammation and coagulation	0.89846
CPB2	Carboxypeptidase B2	Down regulates fibrinolysis by removing C-terminal lysine residues from fibrin that has already been partially degraded by plasmin	0.5268
F2R	Coagulation factor II (thrombin) receptor	High affinity receptor for activated thrombin. May play a role in platelets activation and in vascular development	−0.66526
PROS1	Protein S (alpha)	Anticoagulant plasma protein; it is a cofactor to activated protein C in the degradation of coagulation factors Va and VIIIa. It helps to prevent coagulation and stimulating fibrinolysis	−1.3574
ITGB2	Integrin, beta 2	Are receptors for the iC3b fragment of the third complement component and for fibrinogen	0.91008
PRKCH	Protein kinase C, eta	Serine/threonine-protein kinase that is involved in the regulation of cell differentiation in keratinocytes and pre-B cell receptor	1.3991
RAC1	Ras-related C3 botulinum toxin substrate 1 (rho family, small GTP binding protein Rac1)	Plasma membrane-associated small GTPase which cycles between active GTP-bound and inactive GDP-bound states	0.84209
PDPK1	3-phosphoinositide dependent protein kinase-1	Serine/threonine kinase which acts as a master kinase, phosphorylating and activating a subgroup of the AGC family of protein kinases	0.62861
GNAI2	Guanine nucleotide binding protein (G protein), alpha inhibiting activity polypeptide 2	Guanine nucleotide-binding proteins (G proteins) are involved as modulators or transducers in various transmembrane signaling systems. May play a role in cell division	0.60468
KDM1A	Lysine (K)-specific demethylase 1 A	Component of a RCOR/GFI/KDM1A/HDAC complex that suppresses, via histone deacetylase (HDAC) recruitment, a number of genes implicated in multilineage blood cell development	0.71635

List of differentially expressed top coagulation-related genes “blood coagulation (GO:0007596)” with overlap (45/472) and as the shared signature between Thrombosis, PV and ET individuals from Gene Ontology analysis. Possible roles were extracted from STRING database and the expression values were added from the meta-analysis results.
