# Supplementary material for: Comprehensive Gene expression meta-analysis and integrated bioinformatic approaches reveal shared signatures between thrombosis and myeloproliferative disorders
Source: Sci Rep. 2016 Nov 28;6:37099. doi: 10.1038/srep37099 (PMC5125005; doi:10.1038/srep37099)
Supplement: Supplementary Information [file srep37099-s1.doc]

**SUPPLEMENTARY INFORMATION**

**Comprehensive Gene expression meta-analysis and integrated bioinformatic approaches reveal shared signatures between thrombosis and myeloproliferative disorders**

Prabhash Kumar Jha^1^, Aatira Vijay^1^, Anita Sahu^1^, Mohammad Zahid Ashraf^1*^

^1^Defence Institute of Physiology and Allied Sciences, Delhi, INDIA

**Correspondence:**

Genomics Division, Defence Institute of Physiology and Allied Sciences,

Lucknow Road, Timarpur, Delhi, INDIA,

Postal Code: 110 054, Phone: 91-11-23883190, FAX: 91-11-23914790

E-mail: [mohammadzashraf@gmail.com (M.Z.A)](mailto:mohammadzashraf@gmail.com (M.Z.A)),

**Conflict of Interest Statement:** The authors declare no conflict of interest


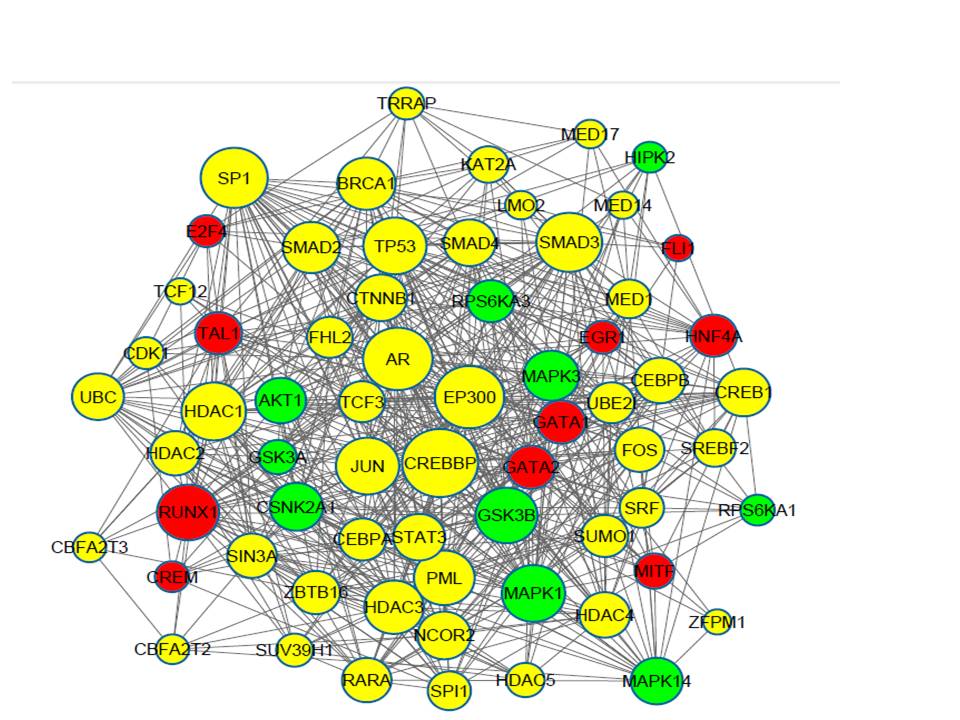


**Figure S1**- Kinase Enrichment Analysis (KEA). Network representation of the top ten enriched Kinases (green) and transcription factors (red) in the meta-analysis of shared DEGs among thrombosis and myeloproliferative diseases. Yellow nodes represents the intermediate proteins in the PPI regulatory network. In the KEA network nodes are interconnected by edges. Nodes size represents the significance of protein based on p-value; bigger the node size higher the significance value.

| **Gene** | **Degree** | **Betweenness centrality (BC)** | **Expression level (Combined ES)** |
| --- | --- | --- | --- |
| **MYC** | 67 | 3771.02 | 1.4034 |
| **FN1** | 67 | 3325.79 | -0.42853 |
| **UBE2I** | 28 | 1215.46 | 0.87053 |
| **IKBKE** | 25 | 688.9 | 0.42291 |
| **ILF3** | 25 | 25.12 | 1.3332 |
| **HNRNPA1** | 23 | 337.96 | 0.6957 |
| **VIM** | 23 | 355.59 | 0.83327 |
| **PARP1** | 22 | 1340.89 | 0.69963 |
| **DDX5** | 22 | 487.15 | 0.48679 |
| **HNRNPM** | 22 | 15.96 | 0.85677 |

**Table S1**- Network based meta-analysis. Top ten genes prioritized based on topological parameters. Expression levels are incorporated in the table from the meta-analysis result.

|  | **Overlap** | ***p-value*** |
| --- | --- | --- |
| **Transcription factors** |  |  |
| FLI1 | 412/5834 | 5.08E-45 |
| HNF4A | 423/6083 | 6.40E-45 |
| RUNX1 | 359/5071 | 4.28E-38 |
| MITF | 371/5578 | 4.81E-34 |
| EGR1 | 370/6204 | 7.57E-25 |
| GATA1 | 196/2601 | 3.31E-22 |
| E2F4 | 215/2998 | 5.85E-22 |
| FOXA2 | 207/2968 | 8.76E-20 |
| SCL | 143/1784 | 2.85E-18 |
| AR | 227/3519 | 6.41E-18 |
| **KINASES** | | |
| MAPK1 | 27/312 | 3.27E-16 |
| MAPK3 | 23/251 | 3.77E-14 |
| HIPK2 | 9/43 | 1.04E-08 |
| AKT1 | 17/256 | 1.92E-08 |
| GSK3B | 25/600 | 2.98E-08 |
| RPS6KA3 | 19/365 | 9.70E-08 |
| MAPK14 | 21/461 | 1.48E-07 |
| GSK3A | 8/44 | 1.93E-07 |
| CSNK2A1 | 22/321 | 2.42E-07 |
| RPS6KA4 | 5/10 | 7.37E-07 |
|  |  |  |

**Table S2**- Top ten TFs and Kinases predicted from the meta-analysis of shared DEGs using Expression2Kinase software.


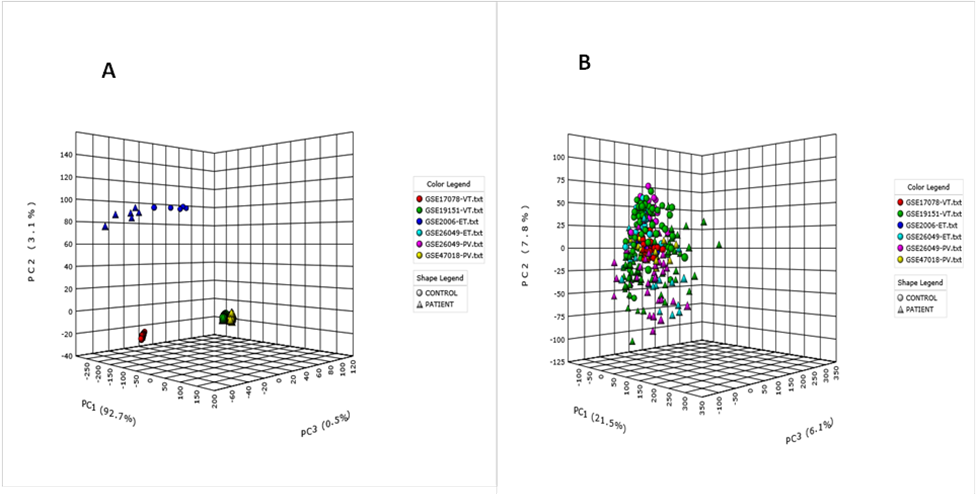


**Figure S2-** Illustration of PCA plots as validation tools for batch effect removal. Plot of principal components: (a) before batch effect removal and (b) after batch effect removal (using Combat method).
